# Development and validation of a nomogram predictive model for cerebral small vessel disease: a comprehensive retrospective analysis

**DOI:** 10.3389/fneur.2023.1340492

**Published:** 2024-01-08

**Authors:** Ning Li, Ying-lei Li, Li-tao Li

**Affiliations:** ^1^Department of Neurology, Hebei Medical University, Shijiazhuang, China; ^2^Department of Neurology, Affiliated Hospital of Hebei University, Baoding, China; ^3^Department of Emergency Medicine, Baoding First Central Hospital, Baoding, China; ^4^Department of Neurology, Hebei General Hospital, Shijiazhuang, China; ^5^Hebei Provincial Key Laboratory of Cerebral Networks and Cognitive Disorders, Hebei General Hospital, Shijiazhuang, China

**Keywords:** cerebral small vessel disease (CSVD), predictive model, nomogram, neuroimaging, blood biochemical markers, retrospective study

## Abstract

**Background:**

Cerebral small vessel disease (CSVD) is a significant contributor to stroke, intracerebral hemorrhages, and vascular dementia, particularly in the elderly. Early diagnosis remains challenging. This study aimed to develop and validate a novel nomogram for the early diagnosis of cerebral small vessel disease (CSVD). We focused on integrating cerebrovascular risk factors and blood biochemical markers to identify individuals at high risk of CSVD, thus enabling early intervention.

**Methods:**

In a retrospective study conducted at the neurology department of the Affiliated Hospital of Hebei University from January 2020 to June 2022, 587 patients were enrolled. The patients were randomly divided into a training set (70%, *n* = 412) and a validation set (30%, *n* = 175). The nomogram was developed using multivariable logistic regression analysis, with variables selected through the Least Absolute Shrinkage and Selection Operator (LASSO) technique. The performance of the nomogram was evaluated based on the area under the receiver operating characteristic curve (AUC-ROC), calibration plots, and decision curve analysis (DCA).

**Results:**

Out of 88 analyzed biomarkers, 32 showed significant differences between the CSVD and non-CSVD groups. The LASSO regression identified 12 significant indicators, with nine being independent clinical predictors of CSVD. The AUC-ROC values of the nomogram were 0.849 (95% CI: 0.821–0.894) in the training set and 0.863 (95% CI: 0.810–0.917) in the validation set, indicating excellent discriminative ability. Calibration plots demonstrated good agreement between predicted and observed probabilities in both sets. DCA showed that the nomogram had significant clinical utility.

**Conclusions:**

The study successfully developed a nomogram predictive model for CSVD, incorporating nine clinical predictive factors. This model offers a valuable tool for early identification and risk assessment of CSVD, potentially enhancing clinical decision-making and patient outcomes.

## Introduction

Cerebral small vessel disease (CSVD) *encompasses* a range of clinical, imaging, and pathological syndromes resulting from various etiologies affecting the small arteries, arterioles, capillaries, venules, and small veins within the brain (1). Prominent radiological manifestations include recent subcortical infarcts, lacunar infarctions, subcortical white matter lesions, cortical surface iron deposition, perivascular spaces, cerebral microbleeds, and microinfarctions (1, 2). CSVD is responsible for approximately a quarter of ischemic strokes, the majority of intracerebral hemorrhages in individuals over the age of 65, and is the leading cause of vascular dementia (3, 4). With the gradual escalation of the burden of CSVD, patients may exhibit symptoms such as cognitive impairment, motor dysfunction, mood disorders, and urinary and fecal incontinence (5). In its early stages, CSVD often presents with no symptoms or only mild symptoms, necessitating a reliance on imaging studies for diagnosis. While cranial magnetic resonance imaging serves as the primary diagnostic tool for CSVD, it comes with a relatively high cost and encounters certain challenges in early detection. In this context, there has been significant focus on predictive models for cerebrovascular diseases, primarily aimed at forecasting disease outcomes and patient prognosis. Despite these advances, there remains a distinct lack of models specifically designed for early diagnosis of CSVD (6–8). Addressing this gap, our study proposes the development of a nomogram predictive model specifically for CSVD diagnosis. This model integrates cerebrovascular risk factors and blood biochemical markers to promptly identify individuals at high risk, enabling early intervention and improved care by healthcare professionals. Such proactive management is crucial in slowing disease progression and enhancing patient outcomes.

## Methods

### Study population and design

This study employed a retrospective design, primarily due to the nature of our approach which involved collecting and analyzing existing case records. Conducted at the neurology department of the Affiliated Hospital of Hebei University from January 2020 to June 2022, the study meticulously gathered data from pre-existing medical records. This approach was chosen as it allows for a comprehensive analysis of already available data, thus providing insights into the patterns and correlations that may not be evident in prospective studies. The retrospective analysis also enabled us to utilize a large sample size, enhancing the statistical power of our findings. The inclusion criteria were carefully designed to ensure a representative sample of the patient population, with particular attention to the completeness and quality of the MRI sequences. The exclusion criteria were set to omit cases with poor-quality MRI images or significant stroke that could impede the accurate assessment of CSVD, as well as patients with severe comorbid conditions that might confound the study results. The patients met the following inclusion criteria: (1) Age >55 years. (2) Complete cranial magnetic resonance imaging (MRI) sequences, including T1-weighted axial, T2-weighted axial, T2-weighted fluid-attenuated inversion recovery (FLAIR), and axial susceptibility-weighted images. Exclusion criteria: (1) Poor MRI image quality or significant stroke that hampers the assessment of CSVD. (2) Presence of severe cardiac diseases (acute myocardial infarction or severe heart failure), severe infections, severe respiratory insufficiency, advanced renal or hepatic diseases, tumors, or any other conditions that may lead to abnormal laboratory results. Potential non-vascular origin of CSVD, such as multiple sclerosis, intracranial tumors, or central nervous system demyelinating diseases. Insufficient clinical or laboratory data. This study, conducted with a retrospective design, strictly adhered to ethical research practices, focusing on the meticulous collection and analysis of pre-existing patient data and records. Emphasizing the privacy and confidentiality of patient data, all records were anonymized prior to analysis, with personal identifiers removed to maintain strict confidentiality. The data handling and analysis process conformed to the guidelines set by data protection regulations, both nationally and internationally, including adherence to the principles outlined in the Declaration of Helsinki. The Institutional Review Board (IRB) of the Affiliated Hospital of Hebei University conducted a comprehensive review of our data protection measures and granted approval (Approval Number: HDFYLL-KY-2023-060), ensuring that the study met the highest standards of patient data security and ethical research practices. This commitment to data security and ethical compliance highlights our dedication to respecting participant privacy while contributing to cerebral small vessel disease research.

### MRI acquisition and assessment

Participants underwent brain MRI using a 1.5T MRI scanner (Siemens, Munich, Germany). The neuroimaging criteria for CSVD in this study included the presence of one or more of the following features: lacunes, white matter hyperintensities (WMH), enlarged perivascular spaces (EPVS), and cerebral microbleeds (CMBs). WMH refers to areas of increased signal intensity on T2-weighted brain images, typically observed symmetrically between the hemispheres. Lacune was defined as a rounded or ovoid lesion of CSF signal measuring 3–15 mm in diameter. CMBs were rounded, hypodense lesions with sizes of 2–10 mm in a susceptibility-weighted image (1). The presence of one or more of these CSVD neuroimaging features was considered as an indicator of the presence of CSVD. The CSVD group included individuals with MRI scans that exhibited one or more CSVD-related features. In contrast, the control group was composed of individuals whose MRI scans showed no evidence of CSVD, and their brain images were considered normal. The assessment of CSVD neuroimaging features was carried out by two experienced neurologists, namely Y. Jiang and N. Li. These neurologists were blinded to the clinical data of the participants. The evaluation was carried out according to the guidelines for reporting vascular changes in neuroimaging (STRIVE) (1, 9).

### Clinical blood biochemistry assessment

We retrospectively collected comprehensive data on the following clinical parameters from the enrolled patients: complete blood count, renal function, electrolyte levels, coagulation profile, random blood glucose, fasting blood glucose, liver function, lipid profile, cardiac enzyme panel, thyroid function tests, and homocysteine levels. In this study, a total of 81 laboratory parameters, encompassing blood glucose, glycated hemoglobin, complete blood count, electrolytes, renal function, coagulation profile, liver function, lipid profile, cardiac enzyme panel, thyroid function tests, and homocysteine, were included. These laboratory parameters were transformed into binary categorical variables based on their respective median or cutoff values.

### Clinical evaluation

Demographic information, including age and sex, as well as medical history, such as hypertension, diabetes, and hypercholesterolemia, were collected. Additionally, information on smoking history, alcohol consumption history, and disease duration was gathered. Hypertension was defined as systolic blood pressure ≥140 mmHg and/or diastolic blood pressure ≥90 mmHg, or as individuals receiving antihypertensive medications. Diabetes was defined as fasting blood glucose levels ≥7.0 mmol/l, OGTT2h levels ≥11.1 mmol/l, or the use of hypoglycemic medications. Hypercholesterolemia was defined as total cholesterol levels or LDL cholesterol levels exceeding the upper limit of the normal range. This study includes two groups of subjects based on the presence or absence of a history of stroke: the Stroke Group, consisting of individuals who have experienced stroke at some point, and the Non-Stroke Group, comprising individuals who have never had a stroke. This study categorized the participants into two groups based on the results of carotid artery ultrasound examination: the Carotid Atherosclerosis Group, comprising individuals with carotid artery ultrasound findings indicating intima-media thickening, plaques, or stenosis; and the Non-Carotid Atherosclerosis Group, consisting of individuals with normal carotid artery ultrasound results.

### Statistical analysis

A cohort comprising 587 patients underwent random allocation into two distinct sets: the training dataset, consisting of 412 individuals, and the validation dataset, consisting of 175 individuals. This allocation adhered to a predetermined ratio of 7:3. In the development of the model, the conversion of continuous variables into categorical ones was adopted, a strategy commonly employed in the literature for risk prediction models to facilitate ease of interpretation and to bolster generalizability. This approach is widely recognized for its clinical applicability and broad acceptance (10, 11). The determination of cutoff points for these continuous variables was guided by clinical insights and mirrored current practices in the literature, as well as established statistical analyses. Categorical variables were presented as frequencies with corresponding percentages (%). To compare the baseline characteristics between the training set and the validation set, we employed statistical tests appropriate for the data types. Specifically, we used the χ^2^ test or Fisher's exact test for categorical variables. In the training set, we applied the Least Absolute Shrinkage and Selection Operator (LASSO) technique to identify the most significant risk factors associated with CSVD. Variables exhibiting non-zero coefficients in the LASSO regression model were chosen for subsequent analysis. In the training cohort, the selected variables were incorporated into a multivariable logistic regression analysis to identify independent clinical predictors associated with CSVD. Subsequently, a nomogram was constructed based on these risk factors in a multivariable analysis. An analysis of the Area Under the Receiver Operating Characteristic Curve (AUC-ROC) was performed to assess the predictive accuracy of the nomogram. Calibration curves were generated to compare predicted probabilities with observed probabilities. Decision Curve Analysis (DCA) was employed to assess the clinical utility of the predictive model. Statistical analyses were conducted using R software version 4.3.0 (http://www.r-project.org/) and IBM SPSS Statistics for Windows, version 26.0 (IBM, Armonk, NY, USA). Two-tailed *p*-values < 0.05 were considered statistically significant.

## Results

### Baseline characteristics

**Figure 1:**
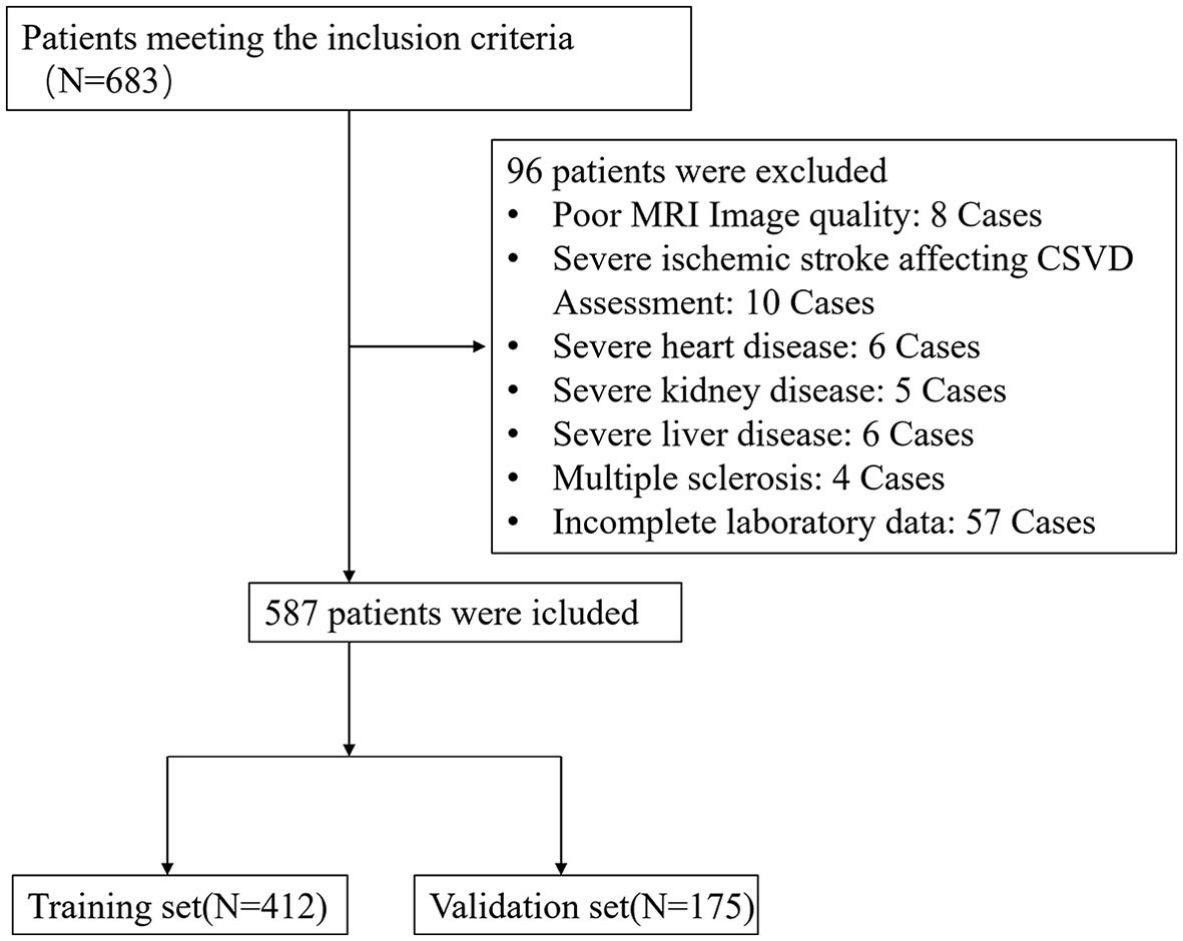
Flow diagram of the selection of eligible patients.

Between January 2020 and June 2022, a total of 683 patients who met the inclusion criteria were initially enrolled in this study. However, 96 patients meeting the exclusion criteria were subsequently removed from the study, resulting in a final cohort of 587 patients eligible for data analysis (Figure 1). The training set consisted of 412 individuals, and the remaining 175 individuals were included in the validating set. The baseline characteristics of the patients in the training and validation set are shown in Table 1.

**Table 1:** Baseline characteristics: comparing CSVD patients with non-CSVD patients.

**Variables**	**Total (*n* = 587)**	**Non-CSVD (*n* = 299)**	**CSVD (*n* = 288)**	***p***
**Gender**, ***n*** **(%)**				< 0.001
Female	307 (52)	186 (62)	121 (42)	
Male	280 (48)	113 (38)	167 (58)	
**Age (years)**, ***n*** **(%)**				< 0.001
≤ 57	170 (29)	104 (35)	66 (23)	
58–63	133 (23)	87 (29)	46 (16)	
64–69	143 (24)	67 (22)	76 (26)	
>69	141 (24)	41 (14)	100 (35)	
**Stroke**, ***n*** **(%)**				< 0.001
No	443 (75)	268 (90)	175 (61)	
Yes	144 (25)	31 (10)	113 (39)	
**Carotid atherosclerosis**, ***n*** **(%)**				< 0.001
No	245 (42)	158 (53)	87 (30)	
Yes	342 (58)	141 (47)	201 (70)	
**Hypertension**, ***n*** **(%)**				< 0.001
No	189 (32)	138 (46)	51 (18)	
Level 1	38 (6)	15 (5)	23 (8)	
Level 2	109 (19)	63 (21)	46 (16)	
Level 3	251 (43)	83 (28)	168 (58)	
**Diabetes**, ***n*** **(%)**				0.039
No	437 (74)	234 (78)	203 (70)	
Yes	150 (26)	65 (22)	85 (30)	
**Hyperlipidemia**, ***n*** **(%)**				0.009
No	382 (65)	179 (60)	203 (70)	
Yes	205 (35)	120 (40)	85 (30)	
**Uric acid (**μ**mol/l)**, ***n*** **(%)**				< 0.001
≤ 4.90	220 (37)	138 (46)	82 (28)	
>4.90	367 (63)	161 (54)	206 (72)	
**Creatinine (**μ**mol/l)**, ***n*** **(%)**				< 0.001
≤ 78	479 (82)	275 (92)	204 (71)	
>78	108 (18)	24 (8)	84 (29)	
**CO**_2_ **binding affinity (mmol/l)**, ***n*** **(%)**				0.003
≤ 27	384 (65)	178 (60)	206 (72)	
>27	203 (35)	121 (40)	82 (28)	
**Total cholesterol (mmol/l)**, ***n*** **(%)**				< 0.001
≤ 4.3	270 (46)	111 (37)	159 (55)	
>4.3	317 (54)	188 (63)	129 (45)	
**Low-density lipoprotein (mmol/l)**, ***n*** **(%)**				< 0.001
≤ 2.7	254 (43)	102 (34)	152 (53)	
>2.7	333 (57)	197 (66)	136 (47)	
**Lipoprotein (a) (mg/L)** ***n*** **(%)**				< 0.001
≤ 317	363 (62)	220 (74)	143 (50)	
>317	224 (38)	79 (26)	145 (50)	
**Albumin to globulin ratio**, ***n*** **(%)**				< 0.001
≤ 1.53	336 (57)	138 (46)	198 (69)	
>1.53	251 (43)	161 (54)	90 (31)	
**Homocysteine (**μ**mol/l)**, ***n*** **(%)**				< 0.001
≤ 20	449 (76)	258 (86)	191 (66)	
>20	138 (24)	41 (14)	97 (34)	
**Monocyte-to-HDL ratio**, ***n*** **(%)**				< 0.001
≤ 0.27	127 (22)	88 (29)	39 (14)	
>0.27	460 (78)	211 (71)	249 (86)	
**White blood cell count (**×**10**^9^**)**, ***n*** **(%)**				0.009
≤ 6.73	294 (50)	166 (56)	128 (44)	
>6.73	293 (50)	133 (44)	160 (56)	
**Monocyte count (**×**10**^9^**)**, ***n*** **(%)**				< 0.001
≤ 0.44	295 (50)	172 (58)	123 (43)	
>0.44	292 (50)	127 (42)	165 (57)	
**Calcium (mmol/l)**, ***n*** **(%)**				0.027
≤ 2.32	310 (53)	144 (48)	166 (58)	
>2.32	277 (47)	155 (52)	122 (42)	
**Fibrinogen (g/l)**, ***n*** **(%)**				0.023
≤ 2.88	296 (50)	165 (55)	131 (45)	
>2.88	291 (50)	134 (45)	157 (55)	
**Apolipoprotein A1 (g/l)**, ***n*** **(%)**				0.001
≤ 1.03	296 (50)	131 (44)	165 (57)	
>1.03	291 (50)	168 (56)	123 (43)	
**Apolipoprotein B100 (g/l)**, ***n*** **(%)**				0.002
≤ 0.79	297 (51)	132 (44)	165 (57)	
>0.79	290 (49)	167 (56)	123 (43)	
**Alanine aminotransferase (U/L)**, ***n*** **(%)**				0.005
≤ 16	303 (52)	137 (46)	166 (58)	
>16	284 (48)	162 (54)	122 (42)	
**Aspartate aminotransferase (U/L)**, ***n*** **(%)**				0.028
≤ 19	337 (57)	158 (53)	179 (62)	
>19	250 (43)	141 (47)	109 (38)	
**Lactate dehydrogenase (U/L)**, ***n*** **(%)**				0.012
≤ 158	307 (52)	172 (58)	135 (47)	
>158	280 (48)	127 (42)	153 (53)	
**Albumin (g/l)**, ***n*** **(%)**				0.001
≤ 39	377 (64)	173 (58)	204 (71)	
>39	210 (36)	126 (42)	84 (29)	
**Globin (g/l)**, ***n*** **(%)**				0.001
≤ 26	340 (58)	193 (65)	147 (51)	
>26	247 (42)	106 (35)	141 (49)	
**Direct Bilirubin (**μ**mol/l)**, ***n*** **(%)**				0.024
≤ 3.60	302 (51)	168 (56)	134 (47)	
>3.60	285 (49)	131 (44)	154 (53)	
**Systemic inflammation response index**, ***n*** **(%)**				0.001
≤ 1.23	293 (50)	169 (57)	124 (43)	
>1.23	294 (50)	130 (43)	164 (57)	
**Neutrophil-to-HDL ratio**, ***n*** **(%)**				< 0.001
≤ 3.97	293 (50)	171 (57)	122 (42)	
>3.97	294 (50)	128 (43)	166 (58)	
**High-density lipoprotein**, ***n*** **(%)**				0.007
≤ 1.13	306 (52)	139 (46)	167 (58)	
>1.13	281 (48)	160 (54)	121 (42)	
**Lymphocyte-to-monocyte ratio**, ***n*** **(%)**				< 0.001
≤ 3.47	294 (50)	129 (43)	165 (57)	
>3.47	293 (50)	170 (57)	123 (43)	

In our study, we compared two groups: the CSVD group and the non-CSVD group. We found significant differences (*P* < 0.05) in 32 out of the 88 analyzed biomarkers between these two groups, while the remaining 56 biomarkers showed no significant differences. The 32 biomarkers with significant differences included: Gender, Age, Stroke, Carotid Atherosclerosis, Hypertension, Diabetes, Hyperlipidemia, Uric Acid, Creatinine, CO_2_ Binding Affinity, Total Cholesterol, Low-Density Lipoprotein, Lipoprotein(a), Albumin to Globulin Ratio, Homocysteine, Monocyte-to-HDL Ratio, White Blood Cell Count, Monocyte Count, Calcium, Fibrinogen, Apolipoprotein A1, Apolipoprotein B100, Alanine Aminotransferase, Aspartate Aminotransferase, Lactate Dehydrogenase, Albumin, Globin, Direct Bilirubin, Systemic Inflammation Response Index, Neutrophil-to-HDL Ratio, High-Density Lipoprotein, and Lymphocyte-to-Monocyte Ratio (Table 1). The other 56 biomarkers showed no significant differences between the two groups including: Fasting Glucose, Platelet Count, Mean Corpuscular Hemoglobin Concentration, Mean Corpuscular Hemoglobin, Mean Corpuscular Volume, Hematocrit, Red Blood Cell Count, Eosinophil Count, Basophil Count, Eosinophil Percentage, Monocyte Percentage, Lymphocyte Percentage, Hemoglobin, Platelet Distribution Width, Mean Platelet Volume, Plateletcrit, Neutrophil Percentage, Neutrophil Count, Lymphocyte Count, Monocyte Count, Platelet Large Cell Ratio, Urea, Sodium, Potassium, Calcium, Magnesium, Serum Phosphate, Prothrombin Time, Prothrombin Time Ratio, International Normalized Ratio, Activated Partial Thromboplastin Time, Thrombin Time, Hemoglobin A1c, Triglycerides, Very Low-Density Lipoprotein, Apolipoprotein E, Creatine Kinase, Creatine Kinase-MB, Alpha-Hydroxybutyrate Dehydrogenase, Alkaline Phosphatase, Gamma-Glutamyl Transferase, Total Protein, Albumin, Globulin, Albumin vs. Globulin Ratio, Total Bilirubin, Direct Bilirubin, Unconjugated Bilirubin, Bile Acid, Triiodothyronine, Thyroxine, Free Triiodothyronine, Free Thyroxine, Thyroid-Stimulating Hormone, and Systemic Immune-Inflammatory Index.

### Variable selection

**Figure 2:**
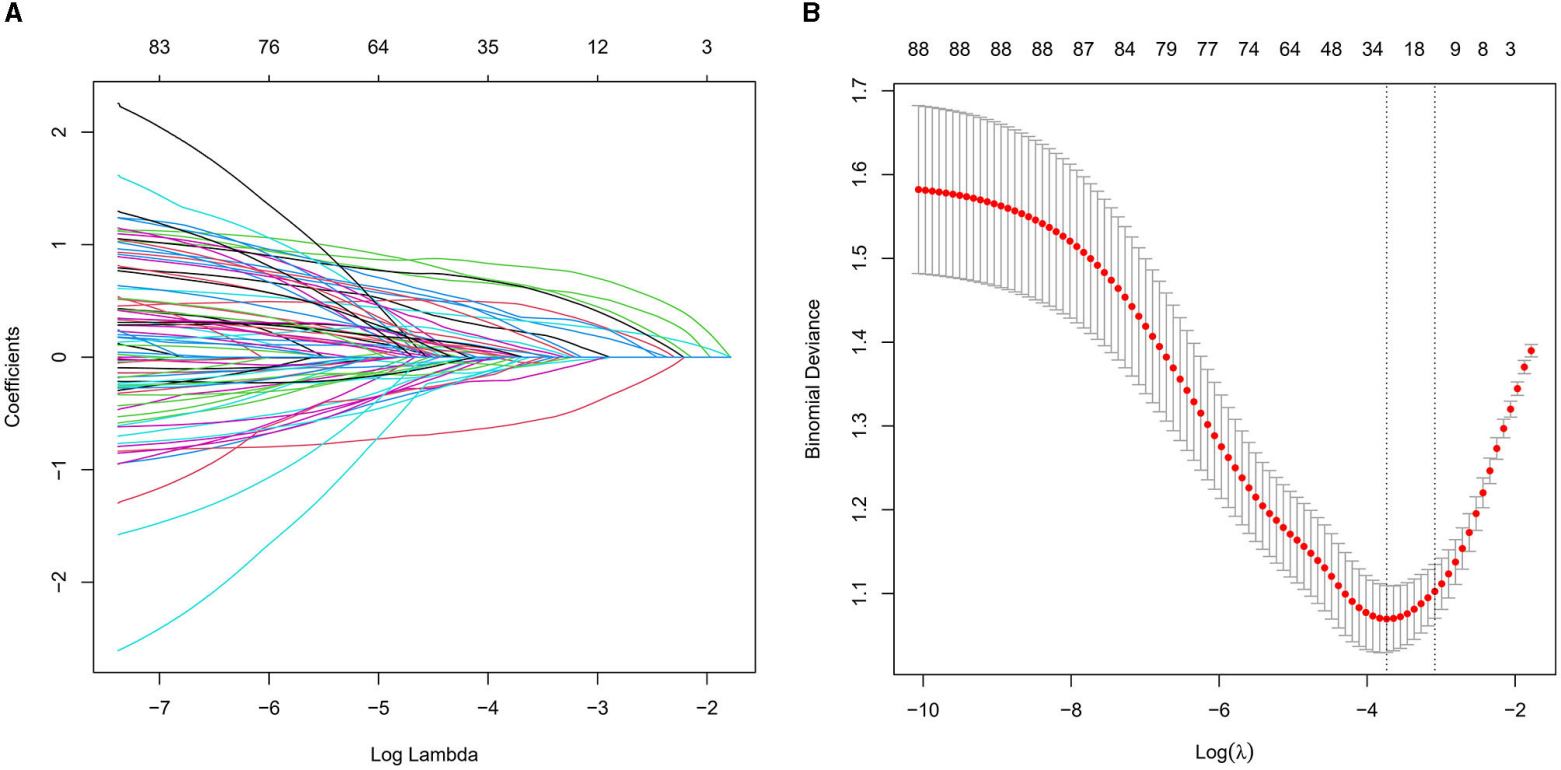
**(A)** LASSO coefficient profiles for CSVD clinical predictors. This figure presents the coefficient profiles of 88 features considered in the LASSO model for predicting the risk of CSVD. The graph displays how each feature's coefficient varies with the log of lambda [log(lambda)], demonstrating the shrinkage effect of the LASSO technique. **(B)** LASSO regression cross-validation results. This figure illustrates the evaluation of model performance under various regularization parameters λ through cross-validation in LASSO regression. A vertical dashed line on the left side represents λmin, which corresponds to the model with the best performance. On the right side, another vertical dashed line denotes λ1SE, representing a slightly sparser model. The numbers of selected variables are annotated above each line.

We collected data covering a total of 88 variables, including age, gender, medical history, and laboratory tests. In our study, the LASSO regression was implemented using the “glmnet package” in R with a 10-fold cross-validation approach to optimize the regularization parameter λ. We selected the value of λ based on the 1SE (one standard error) criterion, which aims at choosing a simpler model with a performance within one standard error of the minimum cross-validation error. This approach helped in striking a balance between model complexity and prediction accuracy, enabling us to identify the most relevant predictors for our model while controlling for overfitting (Figures 2A, B). Through LASSO regression, we selected 12 indicators: Gender, Age, Stroke, Carotid Atherosclerosis, Hypertension, Creatinine, Total Cholesterol, Low-Density Lipoprotein, Lipoprotein(a), Albumin to Globulin Ratio, Homocysteine, and Monocyte-to-HDL Ratio (Table 2). The variables with non-zero coefficients in the LASSO regression model were considered to be related to CSVD.

**Table 2:** Coefficients and lambda.1SE value of the LASSO regression.

**Variable**	**Coefficients**	**Lambda.1-SE**
Gender	0.01578146	0.03784
Age	0.06940418	
Stroke	0.15863094	
Carotid_atherosclerosis	0.04352613	
Hypertension	0.05894357	
Creatinine	0.11182955	
Total cholesterol	−0.0047317	
Low-density lipoprotein	−0.0095456	
Lipoprotein(a)	0.09607666	
Albumin to globulin ratio	−0.0918666	
Homocysteine	0.10097174	
Monocyte-to-HDL ratio	0.06366454	

### Multivariable analyses

In the multivariable logistic regression analysis, we included the 12 variables identified from the LASSO regression. After adjusting for confounding factors, the analysis revealed that nine variables (Gender, Age, Stroke, Carotid Atherosclerosis, Hypertension, Creatinine, Lipoprotein(a), Albumin to Globulin Ratio, and Homocysteine) were significantly associated with the risk of cerebral small vessel disease (*P* < 0.05), as shown in Table 3. The results indicated that these nine variables were independent clinical predictors of CSVD.

**Table 3:** Multivariable logistic regression analysis of clinical predictors of CSVD.

	**B**	**SE**	**OR**	**95% CI**	***Z***	***P***
Gender	0.526	0.233	1.690	1.07–2.67	2.253	0.024
Age	0.376	0.099	1.460	1.21–1.77	3.799	*p* < 0.001
Stroke	1.188	0.266	3.280	1.95–5.53	4.466	*p* < 0.001
Carotid_atherosclerosis	0.443	0.224	1.560	1.10–2.42	1.981	0.028
Hypertension	0.461	0.085	1.590	1.34–1.87	5.451	*p* < 0.001
Creatinine	0.798	0.306	2.220	1.22–4.05	2.606	0.009
Total cholesterol	0.248	0.433	1.280	0.55–2.99	0.573	0.567
Low-density lipoprotein	−0.875	0.433	0.420	0.18–0.97	−2.022	0.063
Lipoproteina	1.121	0.220	3.070	1.99–4.72	5.091	*p* < 0.001
Albumin to globulin ratio	−0.963	0.219	0.380	0.25–0.59	−4.397	*p* < 0.001
Homocysteine	0.728	0.269	2.070	1.22–3.51	2.703	0.007
Monocyte-to-HDL ratio	0.434	0.265	1.540	0.92–2.6	1.637	0.102

This table presents the results of the binary logistic regression analysis, which was conducted on 12 variables initially identified through LASSO regression as potential predictors for CSVD. Out of these, nine variables were retained in the final CSVD diagnostic model, namely (Gender, Stroke, carotid_atherosclerosis, Hypertension, Creatinine, Lipoproteina, Albumin_to_Globulin_Ratio, Homocysteine, and Age). The remaining three variables that were not included in the final model due to P-values > 0.05 are (Total Cholesterol, Low-Density Lipoprotein, Monocyte-to-HDL Ratio). The table details the regression coefficient (B), standard error (SE), odds ratio (OR), 95% confidence interval (95% CI), Z-value, and P-value for each of the nine included factors. The OR values indicate the relative risk associated with each factor, where values >1 suggest an increased risk, and < 1 suggest a decreased risk of CSVD.

### Predictive model development

**Figure 3:**
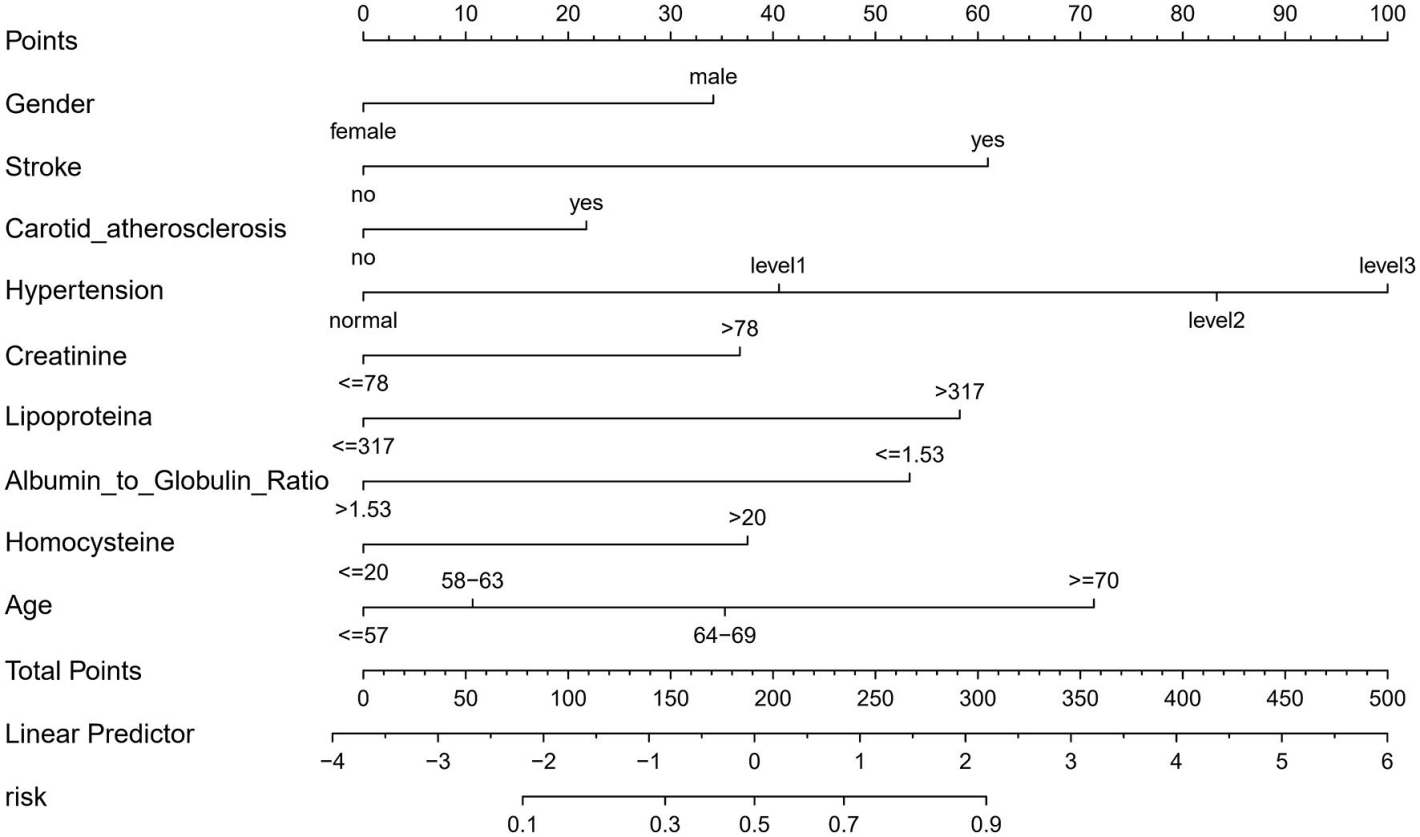
Nomogram for predicting the risk of CSVD. This figure presents a nomogram developed for the early diagnosis of CSVD. The nomogram is constructed based on a multivariable logistic regression analysis, incorporating key clinical predictors identified through the Least Absolute Shrinkage and Selection Operator (LASSO) technique. In this graphical tool, each predictor is assigned a score based on its coefficient in the regression model. The total score, obtained by summing the individual scores for all predictors, can be used to estimate the probability of a patient developing CSVD. This probability scale is provided at the bottom of the nomogram, facilitating its practical application in a clinical setting for risk assessment and early intervention in individuals at high risk of CSVD.

In this study, logistic regression analysis was employed to identify key variables associated with the risk of cerebral small vessel disease (CSVD). Nine variables were selected based on their statistical significance: Gender, Age, Stroke, Carotid Atherosclerosis, Hypertension, Creatinine, Lipoprotein(a), Albumin to Globulin Ratio, and Homocysteine. These variables were then used to construct a nomogram, as depicted in Figure 3. The nomogram operates by assigning point values to each variable based on their calculated beta coefficients, reflecting their proportional prognostic impact. The total points accrued from each variable are then used to estimate the patient's probability of developing CSVD. This probability is derived from the total point's projection on the probability scale provided at the bottom of the nomogram. The inclusion of diverse variables, ranging from biochemical markers like Creatinine and Homocysteine to clinical features such as the presence of Carotid Atherosclerosis, allows for a comprehensive assessment of CSVD risk. This approach enables healthcare providers to make more informed decisions regarding patient care and risk management.

### Nomogram validation

**Figure 4:**
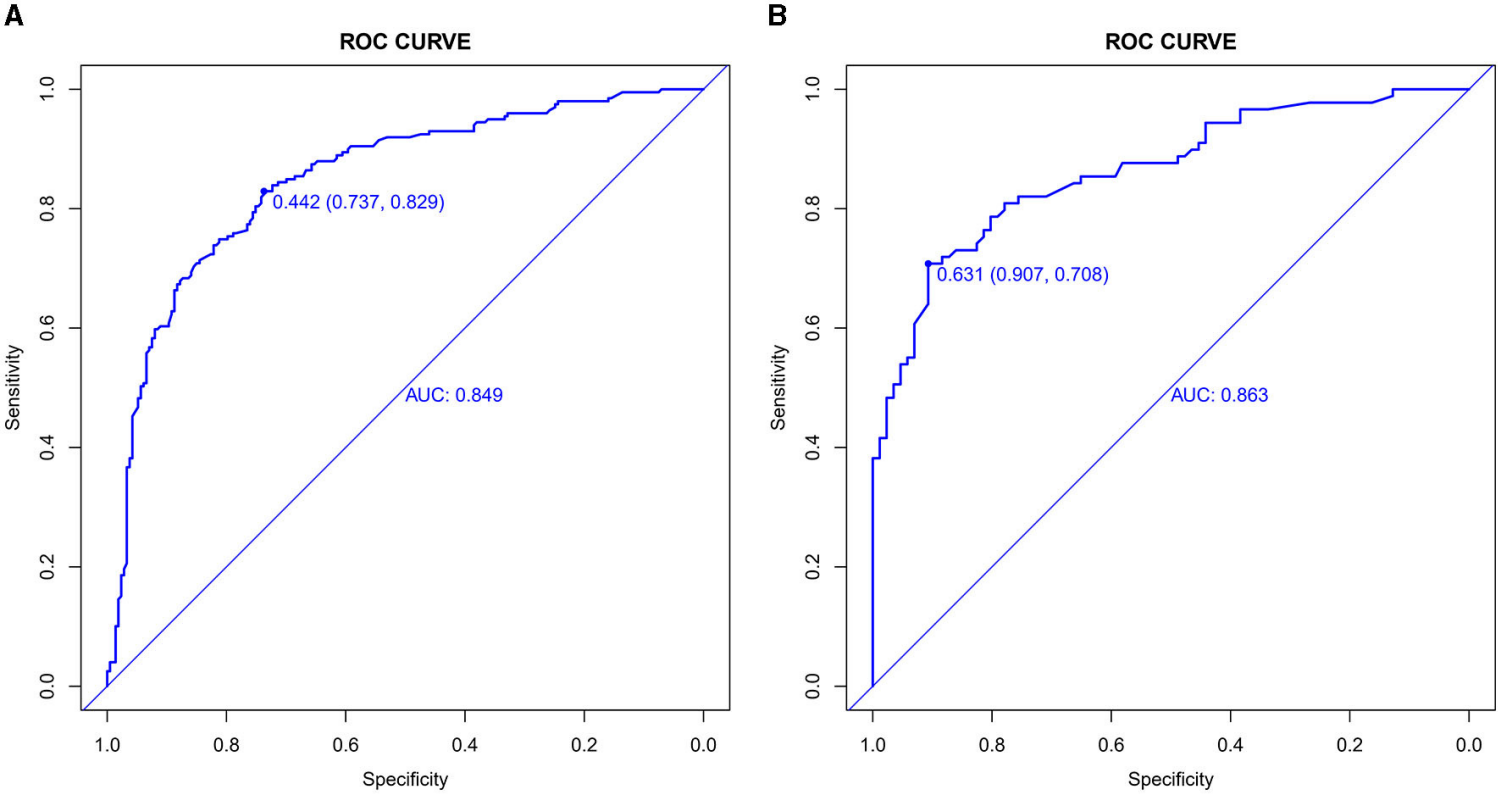
**(A, B)** Receiver operating characteristic (ROC) curves for CSVD predictive model in training set **(A)** and validation set **(B)**. The ROC curves plot the sensitivity against the specificity for various threshold levels. The Area Under the Curve (AUC) value for each set quantifies the overall performance of the model, indicating its capability to distinguish between patients with and without CSVD. A higher AUC value represents better discriminative ability of the model in both training and validation phases.

**Figure 5:**
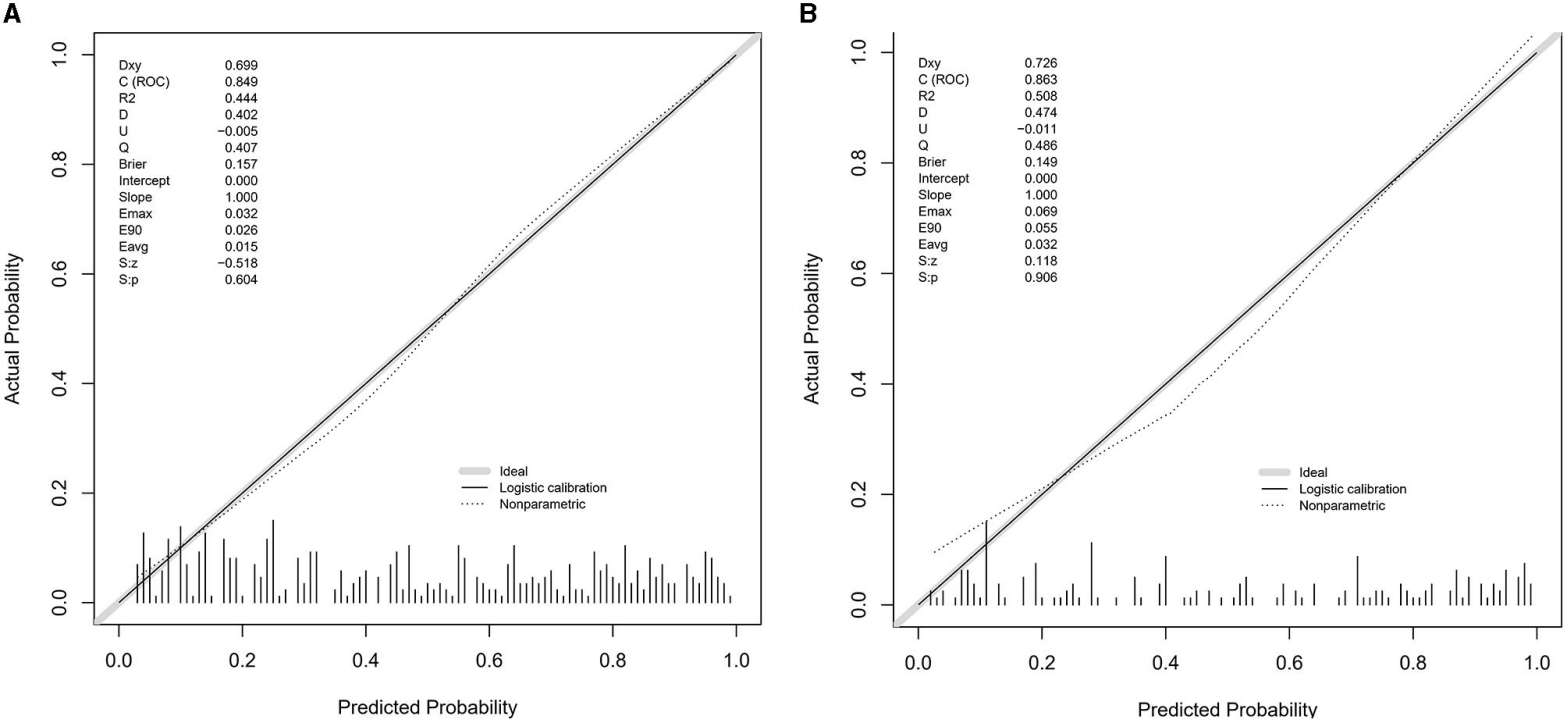
**(A, B)** Calibration plots for the CSVD predictive model using the training set **(A)** and the testing set **(B)**. The calibration plots compare the predicted probabilities of CSVD, as provided by the nomogram, against the actual observed frequencies. Ideally, a model's predictions would align perfectly with the 45-degree diagonal line, indicating a complete match between predicted probabilities and actual occurrences. The closer the points are to this line, the higher the consistency between the model's predictions and the actual outcomes, demonstrating good calibration performance in both sets.

**Figure 6:**
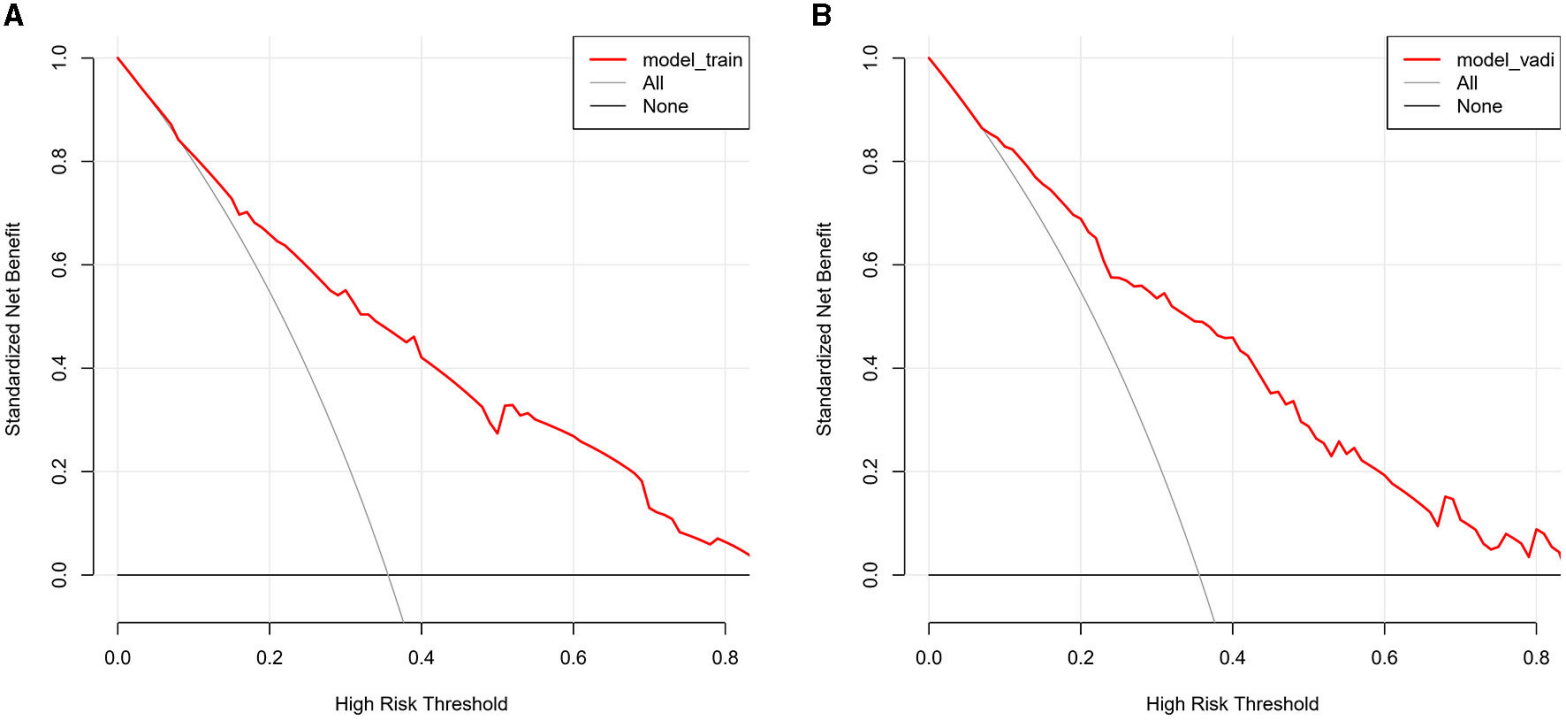
**(A, B)** The decision curve analysis for the CSVD predictive model, applied to the training set **(A)** and the validation set **(B)**. The DCA graphs display the net benefit of using the predictive model at various threshold probabilities compared to two reference strategies: treating all patients or treating none. The horizontal line in each graph represents the scenario where no patients are treated (assuming all are negative), yielding a net benefit of zero. The oblique line represents the opposite extreme, where all patients are treated (assuming all are positive). The curves of the model diverge from these extremes, indicating its clinical utility by providing a balance between the benefits and drawbacks of treatment decisions based on the model's predictions.

The value of AUC-ROC in training set is 0.849 (95% CI: 0.821–0.894). This indicates excellent discriminative ability, meaning the model effectively differentiates between patients with and without CSVD. The value of AUC-ROC in validation set is 0.863 (95% CI: 0.810–0.917). In the validation of the nomogram, we observed high sensitivity and specificity in both the training and validation sets, indicating the model's robust performance in identifying true positive and true negative cases of CSVD. Specifically, in the training set, the sensitivity was 73.7%, and the specificity was 82.9%. In the validation set, the sensitivity further improved to 90.7%, while the specificity was 70.8%. These metrics highlight the model's consistent and strong discriminative power across different datasets (refer to Figures 4A, B for detailed performance metrics; Figures 4A, B). For a detailed breakdown of these performance metrics, including sensitivity, specificity, and other relevant statistical measures, refer to the Supplementary Table 1. Both datasets showed near-ideal slope values of 1.000 and strong C (ROC) indices (0.849 and 0.863, respectively), suggesting reliable predicted probabilities and good model performance (Figures 5A, B). In the Decision Curve Analysis (DCA) graph, the trajectory of the model's curve distinctly deviated from the two extremes, indicative of a substantial clinical benefit. This observation suggests that the model provides significant utility in a clinical setting, as it demonstrates a marked improvement over the baseline strategies represented by the extreme curves (Figures 6A, B).

## Discussion

This study successfully developed a predictive model for cerebral small vessel disease (CSVD) that incorporates nine clinical predictive factors including gender, age, history of stroke, carotid atherosclerosis, hypertension, creatinine, Lipoprotein(a), Albumin/Globulin Ratio, and homocysteine. The comprehensive analysis of these factors provides crucial insights into the risk assessment of CSVD, particularly in the early stages where clinical manifestations are not pronounced. Unlike previous studies focusing on single factors, our multivariate approach offers a more holistic framework for risk assessment. This is particularly vital as CSVD often involves multiple intertwined pathophysiological mechanisms. Additionally, our findings highlight the history of stroke as a robust predictor of CSVD with a significant odds ratio (OR = 3.28). This accentuates the need for heightened vigilance in patients with a stroke history, given their increased risk of developing CSVD. Furthermore, our research corroborates the importance of considering ischemic stroke subtypes when assessing the prognosis of stroke recurrence. Patients with recurrent lacunar infarctions frequently show cognitive impairments, as indicated in previous studies (12). Similarly, in cardioembolic stroke, early recurrent embolization is a crucial predictor of in-hospital mortality (13), emphasizing the varied impact of different stroke subtypes on patient outcomes.

Research has confirmed that cerebral small vessel disease is associated with traditional cerebrovascular risk factors such as hypertension, age, elevated homocysteine levels, and gender (14–16). Furthermore, recent studies have highlighted additional risk factors that may contribute to CSVD, but were not included in our current study. For instance, the relationship between sleep apnea and stroke, as explored in the work by Domínguez-Mayoral et al. (17), points toward sleep apnea as a notable vascular risk factor. Though not a focus of this study, the potential role of sleep apnea in CSVD underscores the complexity of this condition and the necessity of considering a broader range of factors in future research. As our understanding of CSVD evolves, incorporating a wider array of risk factors, including those like sleep apnea, could provide more comprehensive insights into its pathogenesis and aid in the development of more accurate predictive models. Furthermore, we acknowledge the potential importance of cerebral atrophy in the context of CSVD. Although not the primary focus of our study, cerebral atrophy as a manifestation of CSVD has not been adequately characterized in existing literature (18, 19). This limitation of our study is noteworthy, as a deeper understanding of the progression of cerebral atrophy could enhance our exploration of the pathophysiology of CSVD. Another study showed that carotid plaque was associated with the presence of lacunes and larger volumes of white matter hyperintensities (WMH). Increased carotid diameter was associated with lacunes, larger WMH volumes, and perivascular spaces in the basal ganglia. However, carotid intima-media thickness (IMT) and stiffness were not associated with CSVD. These results suggest that carotid atherosclerosis and dilation are linked to CSVD, and non-invasive carotid assessment could be a rational approach for risk stratification of CSVD (20). The results of these studies are consistent with our conclusions. Additionally, our study underscores the potential value of non-traditional biochemical markers such as creatinine, Lipoprotein(a), and the Albumin/Globulin Ratio in the risk assessment of CSVD.

Elevated creatinine, indicative of impaired kidney function, has been increasingly recognized as a contributing factor to cerebrovascular pathologies. This relationship is supported by the study of Fang et al., which demonstrated that chronic kidney disease (CKD) promotes the formation of cerebral microhemorrhages, a key feature of CSVD (21). Furthermore, the systematic review and meta-analysis by Tang et al. reinforce the association between kidney function and brain health, providing additional validation for our model's emphasis on creatinine levels (22). The inclusion of creatinine in our predictive model is not merely a reflection of renal health but may also indicate a broader vulnerability to cerebrovascular conditions. Marini et al. highlighted the genetic overlap between kidney function and cerebrovascular disease, suggesting a shared genetic predisposition that could underlie the association between elevated creatinine levels and CSVD (23). Therefore, the dichotomous classification of creatinine levels in our model serves as a crucial predictor, signifying not just the state of renal function but potentially reflecting the underlying risk for cerebral vascular pathology. This underscores the importance of a holistic approach in assessing CSVD risk, emphasizing the need to consider systemic diseases such as renal impairment.

Lipoprotein(a) is a unique lipoprotein variant, distinguished by its structure and physiological roles, primarily associated with cardiovascular diseases (24, 25). Notably, recent studies have suggested a compelling link between elevated lipoprotein(a) levels and increased risk of CSVD (26, 27). These findings align with our model's emphasis on lipoprotein (a) as a significant predictor, providing a molecular basis for understanding its contribution to CSVD risk. The pathophysiological mechanisms underlying this association can be attributed to lipoprotein(a)'s pro-atherogenic and pro-thrombotic properties, which potentially lead to microvascular damage and, consequently, to the development of CSVD.

Current research indicates that the association between the Albumin/Globulin Ratio (A/G ratio) and cerebral small vessel disease (CSVD) remains unclear. However, studies have highlighted its prognostic value in patients with acute ischemic stroke (AIS). For instance, Wang et al. discovered that lower serum A/G levels were linked to poor functional outcomes and increased all-cause mortality at 3 months and 1-year follow-up in AIS patients. Additionally, lower A/G was independently associated with poor outcomes in Acute Ischemic Stroke Patients undergoing Intravenous Thrombolysis. Our study also finds that lower levels of serum A/G are associated with the occurrence of CSVD. While the A/G ratio is primarily used to assess inflammation and nutritional status, its specific role in cerebrovascular diseases warrants further investigation. Given that CSVD is related to inflammation, Endothelial Dysfunction and vascular dysfunction (3, 28–30), the A/G ratio may indirectly influence physiological processes associated with CSVD.

Our study has several limitations. Firstly, its retrospective design may have introduced selection and information biases. Secondly, the sample, originating from a single medical center, might limit the generalizability of the results. Moreover, our study did not encompass all potential risk factors, possibly overlooking some factors influencing CSVD risk. Additionally, it is important to note that due to the retrospective nature of our study, the sample size was based on all cases meeting the inclusion criteria within a specific time frame. Consequently, we did not perform a priori sample size calculation, as the available data volume typically determines the sample size in such studies. This approach may have implications for the results, and we have considered this aspect in interpreting our findings. Nevertheless, our study provides a potent tool for clinicians to identify individuals at high risk of CSVD during routine examinations. Early intervention in these high-risk groups could improve their long-term health outcomes and potentially slow down or prevent the development of CSVD. Future Research Directions Future research should focus on validating the efficacy of our model, especially in patient populations across diverse demographics and geographical locations. Additionally, exploring other potential biomarkers, such as indicators of inflammation and endothelial function, might enhance the predictive accuracy of the model. Prospective studies should also be considered to better understand the pathophysiological progression of CSVD and the long-term effectiveness of the predictive model.

While our study primarily focused on the impact of acquired risk factors in the development of cerebral small vessel disease (CSVD), including hypertension, diabetes, and arteriosclerosis, we acknowledge the potential role of genetic factors in the pathogenesis of CSVD. Due to the retrospective nature of our study design, the accessibility to genetic data was limited. However, further exploration in this area is essential for future research. Particularly, the interplay between genetic and environmental factors might offer crucial insights in the early diagnosis and personalized treatment strategies for CSVD. Therefore, we suggest in our conclusion that future research should take into account the potential significance of genetic factors in the risk assessment of CSVD, thereby providing more comprehensive guidance for clinical practice.

## Conclusion

In summary, our study provides a valuable tool for the early identification and risk assessment of CSVD, potentially improving clinical decision-making and patient prognosis. Despite some limitations, the prospective applications of this model are promising.

## Data availability statement

The raw data supporting the conclusions of this article will be made available by the authors, without undue reservation.

## Ethics statement

The studies involving humans were approved by the Ethics Committee/Institutional Review Board (IRB) of the Affiliated Hospital of Hebei University, approval number: HDFYLL-KY-2023-060. The studies were conducted in accordance with the local legislation and institutional requirements. Written informed consent for participation was not required from the participants or the participants' legal guardians/next of kin since the research involved a retrospective analysis of pre-existing medical records that were anonymized and de-identified prior to analysis, in accordance with the approved ethical guidelines. This retrospective study design, which does not involve any direct patient interaction or intervention, falls under the category where the requirement for written consent can be waived due to the minimal risk to participants and the use of data collected from records for which consent had already been obtained during the original clinical procedures. The IRB granted a waiver for the requirement of written informed consent.

## Author contributions

NL: Writing – original draft, Writing – review & editing. L-tL: Writing – original draft, Writing – review & editing. Y-lL: Writing – original draft.
