# Supplementary material for: Development and validation of a nomogram predictive model for cerebral small vessel disease: a comprehensive retrospective analysis
Source: Front Neurol. 2024 Jan 8;14:1340492. doi: 10.3389/fneur.2023.1340492 (PMC10801164; doi:10.3389/fneur.2023.1340492)
Supplement: Supplementary file 1 [file Table_1.pdf]

Supplementary Table 1:Detailed Performance Metrics of the CSVD Predictive Model in Training and Validation Sets

Metric	training set	validation set
AUC	0.849	0.863
Sensitivity	0.737	0.907
Specificity	0.829	0.708
Positive Predictive Value	0.754	0.798
Negative Predictive Value	0.793	0.767
False Negative	44	20
False Positive	49	18
Positive Likelihood Ratio	3.44	3.641
Negative Likelihood Ratio	0.293	0.28
